# The lncRNA Jpx participates in testosterone-induced H9c2 cell hypertrophy by targeting the miR-145-5p/Nfatc3 axis

**DOI:** 10.3389/fcell.2025.1620369

**Published:** 2025-08-12

**Authors:** Mingxing Wen, Xinyu Zhang, Litao Tong, Yuhui Chen, Zhengjie Li, Yichen Wang, Can Liu, Jinwei Zhang, Liangpeng Ge, Jing Sun, Mingzhou Li, Xuewei Li, Jideng Ma

**Affiliations:** ^1^ State Key Laboratory of Swine and Poultry Breeding Industry, College of Animal Science and Technology, Sichuan Agricultural University, Chengdu, China; ^2^ Chengdu Public Health Clinical Medical Center, Chengdu, China; ^3^ Bazhong Academy of Agriculture and Forestry Sciences, Bazhong, China; ^4^ Chongqing Academy of Animal Sciences, Chongqing, China

**Keywords:** cardiac hypertrophy, testosterone, Jpx, Mir-145-5p, NFATc3

## Abstract

Cardiac hypertrophy is an adaptive cardiac response to overload. The ensuing decompensation eventually leads to heart failure or sudden death. Testosterone can induce cardiomyocyte hypertrophy, although the underlying mechanism has not been completely elucidated. lncRNAs play a vital role in the regulation of cardiac hypertrophy. Jpx is a newly identified lncRNA highly associated with cardiac hypertrophy, although its specific role in cardiac hypertrophy progres-sion remains unclear. Here, we explore the role and underlying mechanism of Jpx in testos-terone-induced cardiomyocyte hypertrophy. Our results show that Jpx is distinctly upregulated in testosterone-induced hypertrophic H9c2 cells. Overexpression of Jpx strikingly enhances testosterone-induced H9c2 cell hypertrophy. Finally, we demonstrate that Jpx acts as an en-dogenous sponge of miR-145-5p, herein identified as a hypertrophy suppressor, and that forced expression of Jpx downregulates miR-145-5p expression to boost Nfatc3 expression and promote hypertrophy. Additionally, a luciferase assay shows that miR-145-5p is a direct target of Jpx, and overexpression of miR-145-5p counteracts the effects of Jpx overexpression on hypertrophic H9c2 cells. Our findings demonstrate that testosterone can induce Jpx expression and that upregulation of Jpx is involved in testosterone-induced H9c2 cell hypertrophy through the miR-145-5p/Nfatc3 axis. Modulation of these may provide a new approach for tackling cardiac hypertrophy.

## 1 Introduction

Cardiac hypertrophy is an adaptive response of the heart to a loss of contractile quality caused by pressure or volume stress, actin mutation, or previous infarction. It is an independent risk factor leading to a significant increase in the morbidity and mortality of a variety of cardiovascular diseases, such as ischemic heart disease, arrhythmias, heart failure, and cardiomyopathy (Nakamura and Sadoshima, 2018; You et al., 2018; Roth et al., 2017; Dong et al., 2010; Brannan et al., 1990). Cardiac hypertrophy is an increase in heart mass or an enlargement of the heart muscle, which can be broadly divided into two types: physiological hypertrophy and pathological hypertrophy. It is worth noting that in the early stages of cardiovascular disease, both physiological and pathological cardiac hypertrophy are initially adaptive responses to cardiac stress and help improve cardiac function, but long-term persistent cardiac hypertrophy is accompanied by adverse cardiovascular events, including heart failure, arrhythmia, and death (Nakamura and Sadoshima, 2018). Therefore, it is essential to explore new molecular mechanisms related to cardiac hypertrophy. In-depth study of the regulatory mechanism of cardiac hypertrophy will provide new ideas for its prevention and treatment.

Non-coding RNAs (ncRNAs) are mainly divided into long non-coding RNAs (lncRNAs) of more than 200 nucleotides in length, and small non-coding RNAs with less than 200 nucleotides, including microRNAs (miRNAs). They have attracted extensive attention because of their pivotal functions in regulating multiple biological processes by regulating coding RNAs (Brannan et al., 1990; Du and Zamore, 2005). Cardiac hypertrophy is a pathological condition in which lncRNAs play significant roles by acting as regulatory molecules (Liu et al., 2020). They regulate gene expression through a variety of mechanisms, thereby participating in the occurrence and development of cardiac hypertrophy. For example, the long non-coding RNA (lncRNA) Cardiac Hypertrophy Related Factor (Chrf) has been shown to regulate cardiac hypertrophy by targeting miR-489 (Wang et al., 2014; Mably and Wang, 2024). The lncRNA Chaer (cardiac hypertrophy associated epigenetic regulator), which is significantly enriched in the heart, is essential for the development of cardiac hypertrophy (Wang et al., 2016). Therefore, further mining of new lncRNAs related to cardiac hypertrophy and investigation of the function of lncRNAs in cardiac hypertrophy are necessary to better understand the regulation of cardiac homeostasis. In addition, recent studies have found that LncRNA-ITGA2 is an attractive diagnostic and therapeutic target for human proliferative vascular diseases (Guo et al., 2025). Jpx is a newly identified lncRNA that is highly related to cardiac hypertrophy (Mably and Wang, 2024). However, the precise role of Jpx in cardiac hypertrophy is still obscure.

miRNAs are a group of small, highly conserved ncRNAs that negatively regulate gene expression by directly binding to the 3′untranslated region (UTR) of target mRNAs for either translational suppression or mRNA degradation (Shenoy and Blelloch, 2014; Bartel, 2004). miRNAs can play a significant role in the regulation of development, differentiation, proliferation, and apoptosis (Wang et al., 2009; Murtaza et al., 2008; Tan et al., 2008; Chen et al., 2005). Numerous miRNAs influence cardiac hypertrophy, such as miR-1 (Yin et al., 2015; Ikeda et al., 2009), miR-133 (Dong et al., 2010; Singh et al., 2010), and miR-22 (Huang et al., 2013; Xu et al., 2012; Tu et al., 2013). miRNAs themselves are not hypertrophic executioners; they exert their effect through targeting hypertrophic genes. It is therefore necessary to further identify the molecular targets of miRNAs. miR-145-5p is an important regulator of cardiac hypertrophy. It inhibits isoproterenol-induced cardiac hypertrophy by targeting transcriptional regulation of the expression and nuclear transport of the transcription factor GATA6 (Li et al., 2013). However, whether miR-145-5p acts on other target genes and participates in the regulation of cardiac hypertrophy remains to be determined.

The aim of the present study is to explore the role of the lncRNA Jpx in testosterone-induced cardiac hypertrophy and its underlying mechanism of action. We show that Jpx is substantially altered in response to hypertrophic stimulation, while Jpx overexpression enhances testosterone-induced cardiac hypertrophy. Mechanistically, Jpx acts as a competing endogenous RNA (ceRNA) to regulate Nfatc3 (Nuclear factor of activated T cells 3) expression by binding miR-145-5p, subsequently promoting cardiac hypertrophy. Our results reveal a novel model of hypertrophic regulation composed of Jpx, miR-145-5p, and Nfatc3.

## 2 Materials and methods

### 2.1 Cell culture and treatment

The rat myocardial H9c2 cell line was obtained from the Shanghai Institutes for Biological Sciences (Shanghai, China). Cells in Dulbecco’s modified Eagle medium (Gibco™, United States) supplemented with 10% fetal bovine serum (charcoal stripped) (Biological Industries (Bioind), Israel) were grown in an incubator with 5% CO_2_ at 37°C. H9c2 cells were treated with 500 nM testosterone (Sigma-Aldrich, United States) for 48 h to induce vitro cardiac hypertrophy. Control group cells were treated with Absolute ethanol (concentration<0.01%) alone.

### 2.2 Quantitative real-time PCR (qRT-PCR) analysis

Total RNA was extracted from the cells using RaPure Total RNA Micro Kit (Magen, Guangdong, China) according to manufacturer’s instruction. To detect mRNAs and lncRNA expression levels, the PrimeScriptTM RT reagent kit with gDNA Eraser (Takara Bio, Inc., Otsu, Japan) and TB Green® Premix Ex Taq™ II (Takara Bio, Inc.) were used for RT and qPCR, respectively, according to the manufacturer’s protocol. The relative expression levels of related genes were normalized to Gapdh. To detect miRNA expression, Mir-X miRNA First-Strand Synthesis Kit (Takara Bio, Inc., Otsu, Japan) and TB Green® Premix Ex Taq™ II were used for RT and qPCR, respectively, according to the manufacturer’s protocol, and U6 was used as the reference for the miRNA level. The sequences of the primers were synthesized by Tsingke Biotechnology Co., Ltd. (Beijing, China) and are listed in Table 1. The relative gene expression was calculated by the 2^−ΔΔCT^ method.

**TABLE 1:** qRT- PCR primer sequences.

Gene name	Primers for qRT-RCR (5′to 3′)
*ANP*	F-ATCCCGTATACAGTGCGGTG R-GCACCTCAGAGAGGGAGCTA
*BNP*	F-AGCTGCTGGAGCTGATAAGAG R-GGCGCTGTCTTGAGACCTAA
*α-SKA*	F-ATTGTGCACCGCAAATGCTT R-CCCTGCAACCATAGCACGAT
*Nfatc3*	F-TACTGTTTCCCAAGGCCCAG R-GGAGAGAAGCAGTCAGAGCA
*Jpx*	F-GCTGGTCCTATCACAGTAGAGC R-TGGACTACCCTTGAGTCTGGA
*GAPDH*	F-AGTGCCAGCCTCGTCTCATA R-TTCTCAGCCTTGACTGTGCC
*miR-145-5p*	F-GTCCAGTTTTCCCAGGAATCCCT
mRQ 3′primer	included in kit
U6	included in kit

### 2.3 Cell transfection

The lncRNA Jpx overexpression plasmid (pcDNA3.1-Jpx) and the empty pcDNA3.1 vector (negative control) were synthesized by Tsingke Biotechnology Co., Ltd. (Beijing, China). The miR-145-5p mimic and the negative control miRNA (miR-NC) were purchased from RiboBio (Guangzhou, China). Thereafter, following the manufacturer’s instruction, H9c2 cells were transfected with above plasmids using Lipofectamine 3000 (Invitrogen, United States) and HiperFect Transfection Reagent (QIAGEN, Germany) respectively, depending on the experiment. Starving cells for 4 h before transfection, after 48 h of transfection, cells were harvested for subsequent use.

### 2.4 Immunofluorescent staining and cell surface area measurement

H9c2 cells were seeded into 48-well plates. For staining of filamentous actin, cells were washed two times with preheated PBS, and fixed with 4% paraformaldehyde at room temperature for 20 min. Then the cells were washed twice with PBS and incubated with glycine for 5 min. Subsequently, after washing twice using PBS, cells were incubated in PBS containing 0.1% BSA for 15 min. They were then stained with a 5 μg/μL) FITC-conjugated Phalloidin (Sigma, United States) for 45 min at room temperature, and photographed using an inverted fluorescence microscope (Olympus, Japan). Cell surface areas were measured using Image-Pro Plus 6.0 software by measuring 5 randomly selected different fields from three independent experiments, and the average value was used for analysis.

### 2.5 Determinations of protein/DNA ratio

The protein concentration was determined by the BCA method (Beyotime, Shanghai, China), as described by the manufacturer with BSA as a standard. Meanwhile, total RNA-free DNA was extracted using DNA extraction kit (TIANGEN, Beijing, China), and DNA concentration was analyzed using Nanodrop 2000 spectrophotometer (GE Healthcare, United States). The protein/DNA ratio of H9c2 cells was then calculated by the total protein concentration divided with the DNA concentration to estimate potential protein synthesis.

### 2.6 Luciferase reporter assay

Luciferase reporter plasmids (PmirGLO-Jpx-WT and PmirGLO-Nfatc3-WT) were synthesized by Tsingke Biotechnology Co., Ltd. (Beijing, China). Briefly, the wild-type fragments of Jpx and the wild-type 3′-untranslated region (3′-UTR) sequence of Nfatc3 were covered into the pmirGLO vector, respectively, to construct recombinant luciferase reporter plasmids. For the luciferase reporter gene assay, the Hela cells were cultured in 48-well culture plates. Thereafter, the corresponding transfection of above luciferase reporter plasmids with miR-NC or miR-145-5p mimics into Hela cells were processed using Lipofectamine 3000 (Invitrogen). Cells were harvested 48 h after transfection, and luciferase activity was measured using the Dual Luciferase Assay System (Promega Corporation, Madison, WI, United States) according to the manufacturer’s protocol, and normalized to the activity of the Renilla luciferase gene.

### 2.7 Bioinformatic analysis of miR-145-5p and NFATc3 interaction

The potential interaction between miR-145-5p and the 3′UTR of NFATc3 was predicted using miRanda (v3.3a, http://www.microrna.org/microrna/getDownloads.do). The mature sequence of rno-miR-145-5p (GTCCAGTTTTCCCAGGAATCCCT) was obtained from miRBase (http://www.mirbase.org/), and the NFATc3 3′UTR sequence (RefSeq ID: XM_039097846) was retrieved from Ensembl. Analysis was performed with default parameters (alignment score >140, free energy <−20 kcal/mol), identifying a binding site at positions 3605–3653.

### 2.8 Statistical analysis

All experiments were repeated three times. The data were shown as means ± standard deviation (S.D.). The statistical analyses were performed using one-way ANOVA (among at least three groups) or Student’s t-test (between two groups). And *P* value <0.05 was considered to be statistically significant difference. The data were analyzed using GraphPad Prism6.

## 3 Results

### 3.1 Jpx is upregulated in testosterone-induced hypertrophic H9c2 cells

**FIGURE 1:**
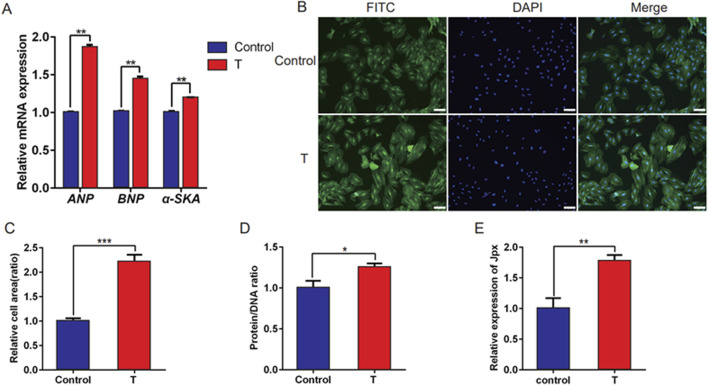
Jpx was upregulated in testosterone-induced hypertrophic H9c2 cells. **(A)** qRT-PCR results of the expression of three hypertrophic markers, ANP, BNP, and α-SKA, in H9c2 cells treated with absolute ethanol (control) or testosterone. **(B,C)** Immunofluorescence staining showing the area of H9c2 cells with absolute ethanol or testosterone treatment. Scale, 200 µm. **(D)** Protein/DNA ratio of H9c2 cells treated with testosterone. **(E)** The Jpx level in absolute ethanol or testosterone-treated H9c2 cells was tested using qRT-PCR. *P < 0.05, **P < 0.01, ***P < 0.001. T, testosterone.

Testosterone has been documented to induce cardiac hypertrophy. However, the underlying molecular mechanisms, including a possible role for lncRNAs, remain to be elucidated fully. Jpx is highly correlated with cardiac hypertrophy (Mably and Wang, 2024), but the expression patterns and precise role of Jpx are still unclear. Therefore, testosterone was applied to construct an *in vitro* model of cardiac hypertrophy. H9c2 cells were treated with 500 nM testosterone for 48 h to induce a cell model of cardiac hypertrophy. qRT-PCR analysis was performed to detect the expression of hypertrophic markers, including ANP, BNP, and α-SKA, in testosterone-treated H9c2 cells. As shown in Figure 1A, testosterone treatment resulted in extremely high expression of ANP, BNP, and α-SKA in H9c2 cells. Meanwhile, we observed that the surface areas of the cells were noticeably dilated, and the protein/DNA ratios, a measure of protein synthesis, were significantly increased (Figures 1B–D). These results indicate that an *in vitro* model of cardiac hypertrophy was successfully established in H9c2 cells upon testosterone treatment. In addition, we further performed qRT-PCR to detect the expression pattern of Jpx, and we observed that Jpx was significantly upregulated in hypertrophic H9c2 cells treated with testosterone compared with the control group (Figure 1E). This result suggests a potential role of Jpx in testosterone-induced cardiac hypertrophy.

### 3.2 Jpx overexpression promotes testosterone-induced cardiomyocyte hypertrophy

**FIGURE 2:**
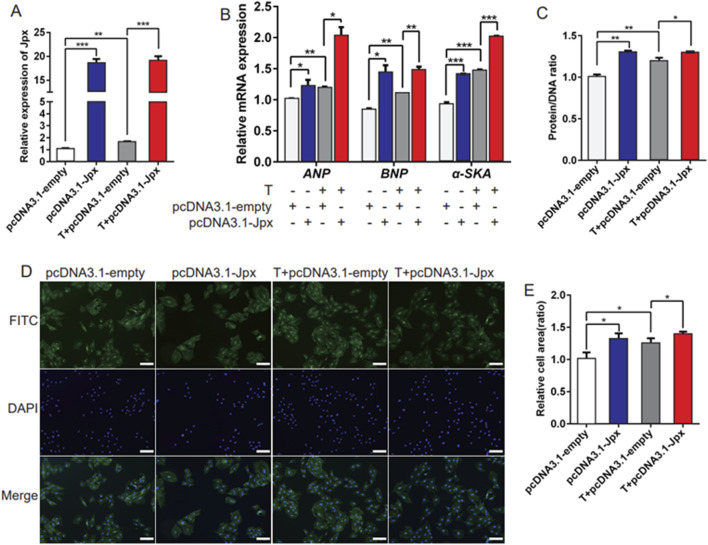
Overexpression of Jpx promotes hypertrophy of H9c2 cells. **(A)** The transfection efficiency of Jpx was detected by qRT-PCR after H9c2 cells were starved for 4 h and then transfected with testosterone, empty pcDNA3.1, or pcDNA3.1-Jpx. **(B)** The relative expression of ANP, BNP, and α-SKA in H9c2 cells after transfection. **(C)** The protein/DNA ratio of H9c2 cells after transfection. **(D,E)** The area of H9c2 cells after transfection was detected by immunofluorescence staining. Scale, 200 μm *P < 0.05, **P < 0.01, ***P < 0.001. T, testosterone.

To explore the role of Jpx in the pathogenesis of testosterone-induced cardiac hypertrophy, pcDNA3.1-Jpx or empty pcDNA3.1 plasmid were transfected into H9c2 cells starved for 4 h. As displayed in Figure 2A, in contrast to the pcDNA3.1-transfected cells, the Jpx level was enhanced in testosterone-induced hypertrophic H9c2 cells after pcDNA3.1-Jpx transfection, indicating that Jpx transfection was successful. Moreover, qRT-PCR analysis demonstrated that Jpx overexpression significantly upregulated the relative mRNA expression of the hypertrophic markers ANP, BNP, and α-SKA in H9c2 cells compared with the pcDNA3.1 group. Similar results were observed in the testosterone treatment group (Figure 2B). Furthermore, consistent with the expression levels of the cardiac hypertrophy markers ANP, BNP, and α-SKA, the protein/DNA ratio of H9c2 cells also increased significantly after Jpx overexpression (Figure 2C). Subsequently, by quantifying the area of H9c2 cells by immunofluorescence staining, it was found that the cell surface area increased significantly after Jpx overexpression compared with the control group (Figures 2D,E). Collectively, this shows that enforced Jpx expression can induce H9c2 cell hypertrophy, and can also enhance the cell hypertrophy induced by testosterone.

### 3.3 Jpx is an endogenous sponge of miR-145-5p

**FIGURE 3:**
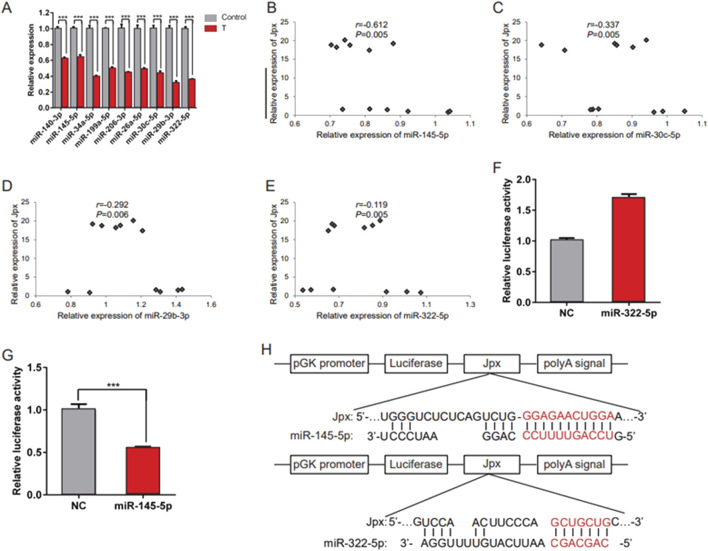
Jpx targets miR-145-5p. **(A)** qRT-PCR results of the expression of candidate miRNAs in absolute ethanol- (control) or testosterone-treated H9c2 cells. **(B–E)** Relative expression correlation analysis between miRNAs (including miR-145-5p, miR-30c-5p, miR-29b-3p, and miR-322-5p) and Jpx in H9c2 cells after Jpx overexpression. **(F,G)** Luciferase reporter assays were conducted to confirm the interaction between Jpx and miR-322-5p/miR-145-5p. **(H)** The structures of the Luc-Jpx-miR-145-5p and Luc-Jpx-miR-322-5p dual luciferase reporter plasmids. *P < 0.05, **P < 0.01, ***P < 0.001. T, testosterone.

Increasing numbers of lncRNAs have been shown to function in cardiac hypertrophy by acting as miRNA sponges. To explore whether Jpx elicits its effect on cardiac hypertrophy through miRNAs, we tested whether one or more miRNAs are a downstream target of Jpx using the online database starBase 2.0. A range of miRNAs are predicted to be potential targets of Jpx, including miR-140-3p, miR-145-5p, miR-34a-5p, miR-199a-5p, miR-206-3p, miR-26a-5p, miR-30c-5p, miR-29b-3p, and miR-322-5p, which are related to the process of cardiac hypertrophy (Hirt et al., 2015; Huang et al., 2014; Li et al., 2017; Sassi et al., 2017). qRT-PCR was performed to analyze the differentially expressed miRNAs in testosterone-induced hypertrophic H9c2 cells. As shown in Figure 3A, these miRNAs were all significantly downregulated depending on testosterone treatment. Moreover, qRT-PCR was performed to detect the differently expressed miRNAs on overexpression of Jpx, and this showed that miR-145-5p, miR-30c-5p, miR-29b-3p, and miR-322-5p were all significantly negatively correlated with the expression of Jpx (Figures 3B–E). We found that only miR-145-5p significantly inhibited the luciferase activity of Jpx, while miR-322-5p had no inhibitory effect (Figures 3F,G). Figure 3H shows the dual luciferase reporter plasmids we constructed. We compared the sequences of Jpx and the four miRNAs above using the online software RNAhybrid, and noticed that Jpx contained binding sites for miR-145-5p and miR-322-5p. Based on these results, we conclude that Jpx exists in the miR-145-5p-assembled RNA-induced silencing complex (RISC) in H9c2 cells.

### 3.4 miR-145-5p inhibits the hypertrophic growth of H9c2 cells

**FIGURE 4:**
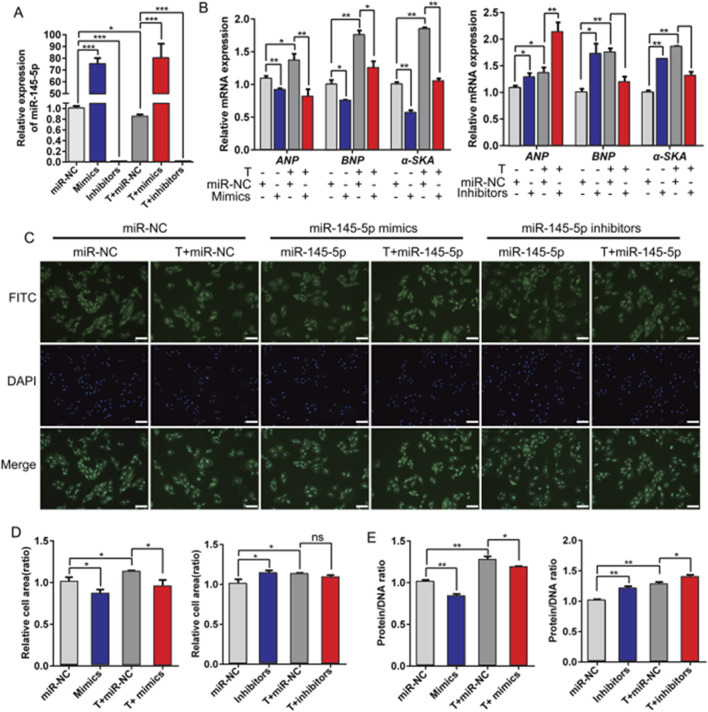
Overexpression of miR-145-5p inhibits testosterone-induced hypertrophy of H9c2 cells. **(A)** qRT-PCR results of miR-145-5p after transfection with testosterone, miR-145-5p mimics, miR-145-5p inhibitors, or miR-NC into H9c2 cells for 48 h. **(B)** The levels of ANP, BNP, and α-SKA in H9c2 cells under different conditions were examined using qRT-PCR. **(C,D)** The area of H9c2 cells after transfection was detected by immunofluorescence staining. Scale, 200 μm. **(E)** The protein/DNA ratio of H9c2 cells under different conditions. *P < 0.05, **P < 0.01, ***P < 0.001. T, testosterone.

Subsequently, we evaluated the role of miR-145-5p in the progression of cardiac hypertrophy. miR-145-5p mimics or miR-145-5p inhibitors were administered in H9c2 cells treated with or without testosterone to induce miR-145-5p overexpression and knockdown, respectively. The transfection efficiency was verified by a significant increase in the miR-145-5p level in the mimic group compared with the miR-NC control group in both the testosterone- and absolute ethanol-treated cells. In contrast, the miR-145-5p levels were overtly suppressed in the inhibitor group (Figure 4A). Consequently, we discovered that miR-145-5p overexpression repressed the baseline expression levels of ANP, BNP, and α-SKA, and downregulated the testosterone-induced increase in expression levels (Figure 4B). Consistently, the cell size of H9c2 cells was also reduced by miR-145-5p, both at the baseline and in response to testosterone (Figures 4C,D). Meanwhile, miR-145-5p also greatly reduced the protein/DNA ratio of H9c2 cells at the baseline and in response to testosterone (Figure 4D). Furthermore, in contrast to the overexpression of miR-145-5p, the inhibition of miR-145-5p increased the baseline mRNA levels of the three hypertrophy marker genes and the baseline area of H9c2 cells, but only upregulated the expression of ANP in response to testosterone (Figures 4B,C). However, miR-145-5p knockdown significantly increased the protein/DNA ratio, both at the baseline and in the testosterone-treated group (Figure 4E). These data demonstrate that miR-145-5p attenuates the hypertrophic response induced by testosterone and plays an inhibitory role in the process of cardiac hypertrophy.

### 3.5 Nfatc3 is a target of miR-145-5p

**FIGURE 5:**
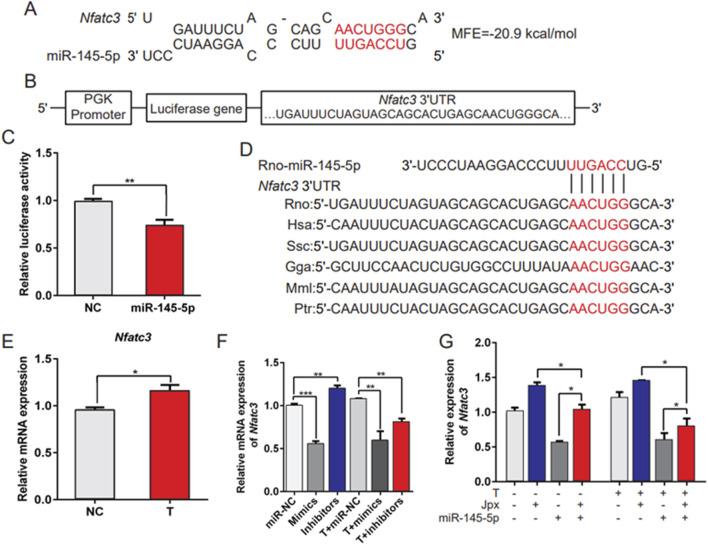
Nfatc3 is a target of miR-145-5p. **(A)** The potential binding sites and minimal free energy (MFE) of miR-145-5p on the 3′UTR of Nfatc3 mRNA. **(B)** The structure of the pmirGLO-Nfatc3 dual luciferase expression vector. **(C)** A luciferase reporter assay was conducted to confirm the interaction between miR-145-5p and Nfatc3. **(D)** The conservation of the miR-145-5p binding site in the Nfatc3 3′UTR among several representative species. **(E)** qRT-PCR results of the expression of Nfatc3 in absolute ethanol- (control) or testosterone-treated H9c2 cells. **(F)** The level of Nfatc3 in H9c2 cells under different conditions was examined using qRT-PCR. **(G)** The expression pattern of Nfatc3 in H9c2 cells was detected by qRT-PCR, in H9c2 cells with negative control (NC) vector, pcDNA3.1-Jpx, and miR-145-5p mimics alone or in combination with pcDNA3.1-Jpx, or miR-145-5p mimics followed by 48 h of testosterone treatment. *P < 0.05, **P < 0.01, ***P < 0.001. T, testosterone; MFE, minimal free energy.

miRNAs negatively regulate gene expression by inhibiting mRNA translation or promoting mRNA degradation. Because miR-145-5p inhibited hypertrophic growth of cardiomyocytes, we anticipated that its target genes would include genes that induced cardiac hypertrophy. The bioinformatics programs TargetScan and miRWalk2.0 were used to identify the target genes of miR-145-5p. Among the predicted potential targets of miR-145-5p, we focused on Nfatc3, because it is the main effector molecule downstream of calcineurin (CaN) in the heart, which is essential for the development of cardiac hypertrophy (Sassi et al., 2017; Altamirano et al., 2009). As shown in Figure 5A, we analyzed the 3′UTR of the Nfatc3 sequence using the bioinformatics program RNAhybrid. The mRNA 3′UTR region consisted of seed sequences matching miR-145-5p. To confirm whether Nfatc3 is a direct target of miR-145-5p, we constructed luciferase reporter gene vectors containing the wild-type 3′UTR of Nfatc3 and performed luciferase reporter assays in HeLa cells (Figure 5B). The results of the luciferase reporter assay demonstrated that ectopic expression of miR-145-5p resulted in notably decreased Nfatc3 luciferase activity (Figure 5C). Furthermore, the binding sites of the Nfatc3 3′UTR and miR-145-5p were highly conserved among different species (Figure 5D). In addition, we observed that Nfatc3 was predominantly upregulated in testosterone-treated H9c2 cells in contrast to absolute ethanol-treated controls (Figure 5E). Moreover, miR-145-5p overexpression significantly restrained the level of Nfatc3 at the baseline and in response to testosterone, while miR-145-5p inhibition only markedly promoted the baseline level of Nfatc3 (Figure 5F). More importantly, the inhibitory effect of miR-145-5p on the level of Nfatc3 was offset by Jpx both at the baseline and in response to testosterone. The level of Nfatc3 was drastically downregulated after transfection of miR-145-5p mimics, and greatly upregulated on Jpx overexpression (Figure 5G). To validate the interaction between miR-145-5p and the NFATc3 3′UTR, we performed bioinformatic analysis using miRanda (v3.3a). Table 2 shows the predicted binding site at positions 3605–3653 in the NFATc3 3′UTR (RefSeq ID: XM_039097846). The alignment of miR-145-5p (GTCCAGTTTTCCCAGGAATCCCT) with the 3′UTR fragment (GGGAAAAGCAGCATTCCTTC) revealed 21 base pairings, including a longest consecutive pairing of 6 bases (AGGAAT with AGCATC), with a free energy of −22.8 kcal/mol and a binding probability of 0.846, indicating a stable interaction. Overall, these results demonstrate that Nfatc3 is a target of miR-145-5p, and that Jpx acts as the competing endogenous RNA (ceRNA) of Nfatc3 by absorbing miR-145-5p in testosterone-induced cardiac hypertrophy.

**TABLE 2:** miRanda Analysis of miR-145-5p and NFATc3 Interaction.

miRNAid	Refseqid	Genesymbol	Start	End	Bindingp	Energy	Number_of_pairings
Rno- miR-145-5p	XM_039097846	Nfatc3	3605	3653	0.846	−22.8	21

### 3.6 miR-145-5p mediates the pro-hypertrophic effect of Jpx on cardiac hypertrophy

**FIGURE 6:**
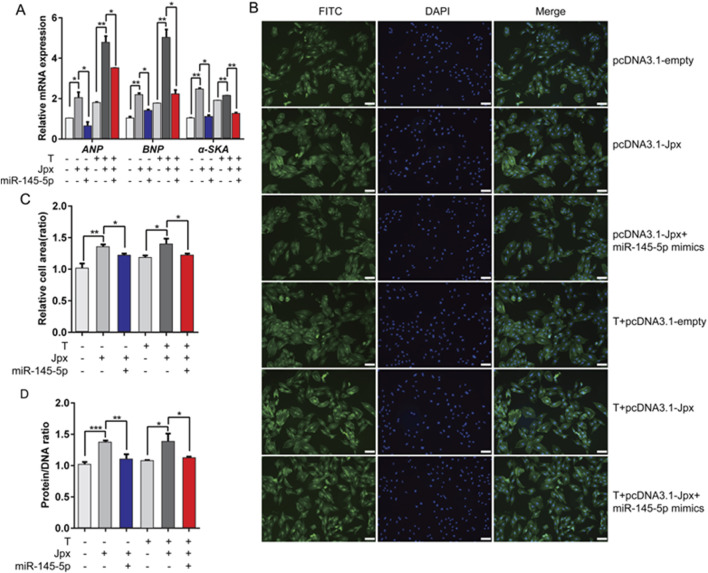
miR-145-5p mediates the pro-hypertrophic effect of Jpx on cardiac hypertrophy. **(A)** The expression levels of ANP, BNP, and α-SKA after H9c2 cells were co-transfected. **(B,C)** The area of H9c2 cells after co-transfection, detected by immunofluorescence staining. Scale, 200 μm. **(D)** Protein/DNA ratio of H9c2 cells after co-transfection. *P < 0.05, **P < 0.01, ***P < 0.001. T, testosterone.

According to the above results, Jpx and miR-145-5p play opposite roles in testosterone-induced cardiac hypertrophy. To confirm whether miR-145-5p is necessary for Jpx-regulated cardiac hypertrophy, we transfected Jpx or vector alone or in combination with miR-145-5p into H9c2 cells, and treated the cells with testosterone for 48 h. As depicted in Figure 6A, co-transfection of miR-145-5p abated the pro-hypertrophic effects of Jpx in the absolute ethanol and testosterone treatment groups, shown by the downregulation of the hypertrophic markers ANP, BNP, and α-SKA. Similarly, co-transfection of miR-145-5p significantly attenuated the increased H9c2 cell area (Figures 6B,C). In addition, we found that co-transfection of miR-145-5p also significantly downregulated the increased protein/DNA ratio (Figure 6D). Taken together, these results suggest that Jpx-induced cardiac hypertrophy is mainly mediated by miR-145-5p.

## 4 Discussion

It has been reported that testosterone can induce cardiac hypertrophy. For example, testosterone activates the mTORC1/S6K1 axis through the IP3/Ca2+ and MEK/ERK1/2 signaling pathways to induce cardiac hypertrophy, and can induce cardiac hypertrophy through CaMKII-mediated activation of the downstream regulatory factor MEF2 (Altamirano et al., 2009; Duran et al., 2017). However, there have been no relevant reports on whether non-coding RNAs are involved in regulating androgen-induced cardiac hypertrophy. Increasing evidence points toward lncRNAs as regulators of cardiac hypertrophy, it regulates gene expression through mechanisms such as the competitive endogenous RNA (ceRNA) network (Han et al., 2017; Viereck et al., 2016). Although lncRNA Jpx was initially studied in the field of X-chromosome inactivation (Oh et al., 2025), its role in cardiovascular diseases remains to be thoroughly explored. Recent studies suggest that Jpx may influence cellular processes by sponging miRNAs, but its mechanisms in cardiac hypertrophy have not been previously reported (Hirt et al., 2015). The lncRNA Mhrt is closely related to cardiac hypertrophy (Zhang et al., 2020), and Chast (cardiac hypertrophy-associated transcript) is a pro-hypertrophy lncRNA, which affects cardiac hypertrophy by improving cardiac remodeling (Viereck et al., 2016). The lncRNA MAGI1-IT1 regulates AngII-induced H9C2 cell hypertrophy via sponge miR-302e (Zhang et al., 2020). The lncRNA ROR facilitates cardiac hypertrophy by combining with miR-133 (Jiang et al., 2016). Herein, we report that treatment with 500 nM testosterone induced hypertrophic phenotypes in H9c2 cells, and such hypertrophic cells showed distinct upregulation of Jpx, a lncRNA correlated with cardiac hypertrophy (Song et al., 2016). Furthermore, we demonstrated that forced expression of Jpx enhanced hypertrophic symptoms of H9c2 cells in both the absolute ethanol and testosterone treatment group.

Recently, an increasing number of studies have shown that lncRNAs exert their functions in disease through regulating gene expression via a variety of mechanisms. Of these, the ceRNA network is the main mechanism that modulates gene expression at the post-transcriptional level (Marchese et al., 2017; Tay et al., 2014). The ceRNA network has also been widely reported in cardiac hypertrophy. For instance, the lncRNA MIAT promotes cardiac hypertrophy by regulating miR-93/Tlr4 (Li et al., 2018). The lncRNA Plscr4 blocks the development of cardiac hypertrophy by targeting the miR-214/Mfn2 axis (Lv et al., 2018). The lncRNA H19-miR-675-CaMKIIδ axis negatively regulates the progression of cardiac hypertrophy (Liu et al., 2016). Here, we identified that Jpx acts as an endogenous sponge of miR-145-5p and suppresses the relative expression of miR-145-5p, which is similar to previous reports on the regulation of cardiac hypertrophy by the ceRNA network (Fang et al., 2020; Zhou et al., 2018; Zhang et al., 2019). Forced expression of miR-145-5p partially alleviated the promoting effect of Jpx overexpression on testosterone-induced cardiac hypertrophy, indicating that Jpx controls the development of testosterone-induced cardiac hypertrophy by acting as a sponge for miR-145-5p. miR-145-5p is an important regulator of cardiac hypertrophy, in contrast, miR-145-5p is a well-established inhibitor of cardiac hypertrophy, suppressing pro-hypertrophic pathways by targeting genes such as GATA6 and IGF1R (Li et al., 2013; Lin et al., 2021). This study confirms that miR-145-5p is downregulated in testosterone-induced cardiac hypertrophy, and functions as a suppressor in absolute ethanol- and testosterone-induced myocardial hypertrophy.

Testosterone promotes cardiac hypertrophy through multiple signaling pathways, including mTORC1/S6K1, CaMKII/MEF2, and calcineurin-NFAT pathways, which activate pro-hypertrophic gene expression and protein synthesis (Altamirano et al., 2009; Duran et al., 2017; Wilkins and Molkentin, 2004). Specifically, testosterone may enhance cardiomyocyte protein synthesis by activating mTORC1/S6K1 signaling (Liu et al., 2025), while the CaMKII/MEF2 and calcineurin-NFAT pathways drive the transcriptional activation of hypertrophy-related genes such as ANP and BNP (Duran et al., 2017). This study unveils a novel Jpx/miR-145-5p/Nfatc3 regulatory axis, wherein lncRNA Jpx acts as a competitive endogenous RNA (ceRNA) to sponge miR-145-5p, thereby upregulating Nfatc3 expression and promoting testosterone-induced hypertrophy in H9c2 cells (Figures 5, 6).

Unlike traditional pathways reliant on protein kinase/phosphatase signaling, the Jpx/miR-145-5p/Nfatc3 axis operates through a non-coding RNA network, offering a unique regulatory mechanism. This axis integrates testosterone signaling with epigenetic regulation: Jpx fine-tunes Nfatc3 activity by modulating the known hypertrophy inhibitor miR-145-5p (Zhou et al., 2021). The biological significance of this finding lies in its first revelation of lncRNA’s novel role in testosterone-mediated cardiac hypertrophy, expanding the molecular framework of androgen-driven cardiac remodeling. Compared to interventions such as mTOR or calcineurin inhibitors, which have broad cellular effects (Chiasson et al., 2017), targeting Jpx or miR-145-5p may enable selective regulation of the hypertrophic response without interfering with other physiological functions of testosterone.

As an important method of epigenetic regulation, miRNAs recognize the 3′UTR of target gene mRNA through the 5′terminal seed sequence, and form a complex through specific complementary base pairing to promote target gene degradation or inhibit translation, thereby regulating gene expression at the post-transcriptional level (McDaneld, 2009). Nfatc3 is a member of the NFAT family. NFAT is an effective regulator of hypertrophic gene transcription, which is closely related to the cardiac hypertrophy signaling pathway mediated by the calcineurin (CaN) mechanism (Molkentin et al., 1998). Increased levels of intracellular Ca2+ can activate CaN, and activated CaN dephosphorylates transcription factors of the NFAT family and translocates them to the nucleus, promoting the binding of NFAT to DNA and its interaction with other transcription factors, thereby activating genes related to cardiac hypertrophy and ultimately leading to cardiac hypertrophy (Wilkins and Molkentin, 2004). In our study, we identified that Nfatc3 is a direct target of miR-145-5p, and the levels of Nfatc3 were also tested and found to be increased in testosterone-induced H9c2 cells. Given that Nfatc3 is the main transcription factor that mediates the signal transduction of cardiac hypertrophy, this study confirms that the lncRNA Jpx participates in testosterone-induced cardiac hypertrophy through the miR-145-5p/Nfatc3 axis. In future, further study is required to determine whether Jpx plays a regulatory role in the occurrence and development of cardiac hypertrophy through the calmodulin signal transduction pathway.

Testosterone exerts a pronounced “double-edged sword” effect on the cardiovascular system. This study demonstrates through the H9c2 cardiomyocyte model that testosterone induces a hypertrophic phenotype via the lncRNA Jpx/miR-145-5p/Nfatc3 axis (Figures 1, 5, 6), consistent with previous reports of testosterone activating pro-hypertrophic pathways such as mTORC1/S6K1 and CaMKII/MEF2 (Altamirano et al., 2009; Duran et al., 2017). Notably, clinical studies indicate that low testosterone levels are associated with increased cardiovascular risks, such as heart failure and atherosclerosis, suggesting a potential protective role in specific populations (Gencer et al., 2021). This paradoxical phenomenon poses challenges for clinical decisions regarding testosterone replacement therapy (TRT): while TRT can improve muscle function and metabolic parameters in patients with hypogonadism (Choi et al., 2022), excessive supplementation may exacerbate cardiovascular burden by enhancing hypertrophic responses.

In summary, we demonstrated that Jpx is upregulated and miR-145-5p is downregulated in testosterone-induced H9c2 cells. Mechanistic analysis suggested that Jpx overexpression facilitates testosterone-induced cardiac hypertrophy by regulating the miR-145-5p/Nfatc3 axis, providing new insights for understanding the function of lncRNAs in the pathogenesis of cardiac hypertrophy. At the mechanistic level, we have demonstrated for the first time that Jpx relieves the suppression of the key hypertrophy-related transcription factor Nfatc3 by sponging miR-145-5p, thereby establishing a novel Jpx/miR-145-5p/Nfatc3 regulatory axis. However, this study validated this molecular mechanism only at the cellular level, and whether it contributes to *in vivo* cardiac hypertrophy remains unclear, necessitating the development of *in vivo* cardiac hypertrophy models to further validate these findings. Additionally, the expression of the Myh7 marker and Nfatc3 protein was not assessed, nor were Jpx knockdown experiments conducted. Future studies are needed to further elucidate the mechanisms by which lncRNAs regulate androgen-induced cardiac hypertrophy, validating the Jpx/miR-145-5p/Nfatc3 axis in primary cardiomyocytes and animal models to enhance its translational significance. This study provides new insights into the investigation of androgen-regulated cardiac hypertrophy.

## 5 Conclusion

In conclusion, this study reveals the significant role of the long non-coding RNA (lncRNA) Jpx in testosterone-induced cardiac hypertrophy. We demonstrated that testosterone treatment upregulates Jpx in H9c2 cardiomyocytes, where it acts as a competing endogenous RNA (ceRNA), sponging miR-145-5p. This interaction leads to the upregulation of Nfatc3, a key transcription factor involved in the hypertrophic signaling pathway. Overexpression of Jpx exacerbates hypertrophic responses, as evidenced by increased expression of hypertrophic markers and enlarged cell surface areas. Importantly, the suppressive effects of miR-145-5p on hypertrophy were counteracted by Jpx, suggesting that miR-145-5p mediates the hypertrophic effect of Jpx through the miR-145-5p/Nfatc3 axis. These findings contribute to our understanding of the molecular mechanisms underlying testosterone-induced cardiac hypertrophy and highlight potential therapeutic targets for mitigating hypertrophic growth in cardiac disease. Future studies should explore the *in vivo* implications of these findings and the broader role of lncRNAs in regulating cardiac pathologies.

## Data Availability

The original contributions presented in the study are included in the article/supplementary material, further inquiries can be directed to the corresponding author.
